# Comparison of Gastric Cancer Models Using Different Dimensions In Vitro

**DOI:** 10.1002/cnr2.70401

**Published:** 2025-11-27

**Authors:** Wenhui Zheng, Yubiao Lin, Lulin Ji, Lihua Feng, Xin Fan, Zhigao Zheng, Yingqin Gao, Kaida Huang, Guoqin Qiu, Yide Chen, Fanghong Luo, Shuitu Feng

**Affiliations:** ^1^ Oncology Department Xiamen Haicang Hospital Xiamen China; ^2^ School of Basic Medicine Central South University Changsha China; ^3^ Chenggong Hospital Affiliated to Xiamen University Xiamen University Xiamen China; ^4^ Cancer Research Center, School of Medicine Xiamen University Xiamen China; ^5^ Department of Medical Oncology Fudan University Shanghai Cancer Center Xiamen Hospital Xiamen China

**Keywords:** drug test, gastric cancer, organoid, spheroid, viability assay

## Abstract

**Background:**

Gastric cancer (GC) is one of the most common cancers worldwide due to its late stage of diagnosis. More efficacious models are required to serve as experimental representatives to enhance the effectiveness of various drugs, select suitable treatment regimens for patients, and further investigate the molecular pathological mechanism of GC.

**Aims:**

This study utilized four GC cell lines and patient‐derived tumor cells (PDTCs) to explore the advantages and disadvantages of the 2D, spheroid, and organoid models, reveal the growth characteristics, drug sensitivity differences, and potential mechanisms of GC cells in different culture models, so as to promote the development of GC models.

**Methods and Results:**

A series of experimental approaches were employed, encompassing but not limited to cell growth assessments, drug sensitivity assays, and RNA‐sequencing analyses. We demonstrated that the 3D models are more like human tumor tissues in terms of tissue structure and spatial structure. Notably, there are also differences between different 3D models. The results clearly indicate that the sizes of organoids and spheroids differ significantly. The spheroid model had the lowest growth rate among the three models. However, the organoid model achieved the highest cell growth rate among the three models. This may be because the organoid showed significant PI3K/PTEN signaling pathway activation and low expression of the apoptosis‐related protein cleaved PARp. Through RNA‐sequencing analysis, we found that the biosynthesis and metabolism of the 3D model were higher than those of the 2D model, which may be one of the reasons for the drug resistance of the 3D model. The spheroid model had the lowest drug sensitivity among the three models. We found that the spheroid model had higher expression of metabolic‐related molecules, followed by the organoid model and then the 2D model.

**Conclusion:**

Our results show a gap among the three models in the growth characteristics, tissue structure, hypoxia, anti‐apoptotic features, signal pathway expression level, and drug sensitivity evaluation of GC cells. These results will further promote the development of GC models.

**Abbreviations**
2D: two‐dimensional
CTG: CellTiter‐Glo
EMT: epithelial‐mesenchymal transition
GC: gastric cancer
GEMT: genetically engineered mouse tumor model
IHC: immunohistochemistry
PBMC: peripheral blood mononuclear cell
PDTCs: patient‐derived tumor cells
PDTX: patient‐derived tumor transplantation
PDX: patient‐derived xenograft

## 1 Introduction

Gastric cancer (GC) is the fourth most common cancer globally, with more than 1 million new cases estimated each year [1, 2, 3]. Considering that GC patients are often diagnosed at a late stage, have low survival rates, and that the clinical development of oncology drugs is costly and time‐consuming [3, 4], there is a need to explore more efficient models as an alternative to human experimentation. Therefore, we assessed whether organoid and spheroid models were more suitable as preclinical platforms by conducting different‐dimensional culture models.

The main advantages of the 2D model are easy maintenance in culture, widely used, and highly repeatable [5]. Non‐adherent surface culture is one of the scaffold‐free methods, in which the cell suspension is added to the ultra‐low adsorption culture plate to make the cells gather and form spheroids [6]. In addition, spheroids successfully replicate the intercellular interactions under non‐adhesive conditions [7, 8]. The organoid model is a process in which cells are arranged into an ordered structure on a specific matrix and then grow to complete autologous tissue [9], with biodiversity more similar to that of tumors in vivo [10, 11, 12]. Thus, the 3D model may enhance our understanding of cancer biology, facilitate the search for new treatments, and bring us closer to the goal of personalized medicine.

**FIGURE 1:**
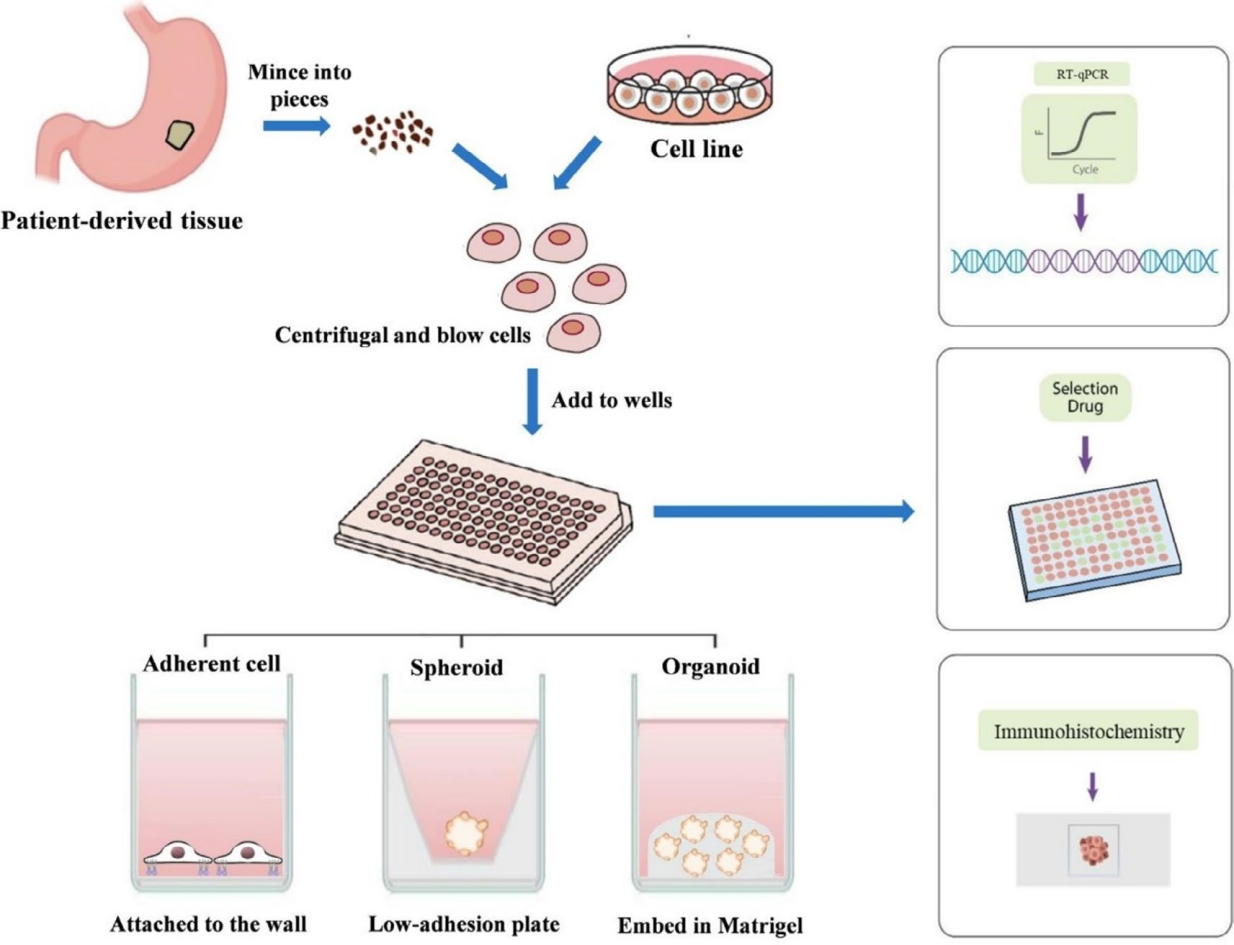
Experimental procedure. GC cell lines and patient‐derived cancer tissue cells were selected for treatment. 96‐well plates were used for the whole process, which were divided into ordinary adherable plates, scaffold‐free plates, and ordinary plates treated with Matrigel.

We present the design of the experiments and compare the growth and morphological characteristics of the different models (Figure 1). In the study, we compared different models by examining the growth characteristics, tissue structure, hypoxia, anti‐apoptotic features, signal pathway expression level, and drug sensitivity. We further performed RNA sequencing to explore the differences among the models at the molecular level.

The cell viability assay showed that the spheroid model had meager proliferation rates and a slower metabolic rate. Meanwhile, the organoids and 2D model had a fast growth rate. The drug sensitivity test showed that the formation of dense multicellular spheroids and organoids in 3D culture plays a role in determining the sensitivity of GC cells to 5‐FU and oxaliplatin. The H&E result showed that the organoid and spheroid models had cells firmly in contact with each other, presenting a more intricate structure. The IHC and western‐blotting experiments also showed that 3D culture promotes cell–cell interactions. The hypoxia experiment showed that the hypoxia status in spheroids and organoids may be one of the causes of their drug resistance. For the observation of 3D models, our results clearly indicate that the sizes of organoids and spheroids differ significantly. The size factors play a crucial role in characteristics like drug resistance and growth rate. Therefore, they could be one reason for the differences in the resistance and growth rates of organoids and spheroids. We attempt to elaborate on the mechanisms of their drug resistance at the molecular level. The phosphatase and tensin homolog (PTEN)–phosphatidylinositol‐3‐kinase (PI3K)/AKT pathway plays an important role in malignant tumor development, whereas PTEN primarily inhibits the PTEN–PI3K/AKT pathway by acting as a lipid phosphatase on PIP3 [13]. We found that the organoid model had the lowest expression level of cleaved PARP and the higher expression level of the PTEN–PI3K pathway. The PTEN–PI3K activation of the signaling pathway implies that cells within the organoid model are in a state of active growth. Matrix metalloproteinase (MMP)‐2, MMP‐9, E‐cadherin, and vimentin are correlated with the development, invasion, and metastasis of cancers [14]. E‐cadherin participates in cellular adhesion and epithelial‐mesenchymal transition (EMT) [15]. We found that MMP2 and MMP9 were substantially expressed in all models, while the expression of E‐cadherin was low in all models, especially in the organoid model. Our results suggested that the 3D models induced cell differentiation more effectively than the 2D model, possibly because the former promoted cell–cell interactions. This characteristic might be one of the causes of its drug resistance. RNA sequencing analysis showed that PTEN, BCL2, CSF1, EPOR, PIK3CD, and FGFR1, which are related molecules of the PTEN–PI3K signaling pathway, were highly expressed in the organoid model. Moreover, RNA sequencing analysis showed that the spheroid model had higher expression of metabolic‐related molecules, followed by the organoid model and then the 2D model.

By comparing the differences between these models, we have promoted the development of preclinical models. This effort has further enhanced the reliability and predictability of research results, reduced the need for animal testing and related ethical concerns, accelerated the drug development process, deepened our understanding of tumor biology, and advanced the field of personalized medicine. We believe that cell line studies could explain part of the cause of the development of the disease. However, the sensitivity of cell lines to drugs is not entirely representative or predictive of patient response due to tumor heterogeneity. In summary, our findings offer compelling support for the logical selection of more personalized clinical research models.

## 2 Methods

### 2.1 Processing of GC Tissues

The GC samples were collected at Haicang Hospital (Xiamen, China); the Haicang Hospital Ethics Committee approved both. Written informed consent was obtained from the patients and/or their authorized representatives and none of the patients received chemotherapy before surgery. The tissue was washed with 1 × PBS solution containing 1% penicillin–streptomycin (Gibco) on the same day after removal. Tumor tissue of 0.5–2 cm^2^ size was removed into a culture dish containing tissue dissociation solution (Gibco) and cut into fragments of 1–2 mm^2^ on ice. The filtrate containing cell masses and single cells was filtered through a 70 μm filter for tissue homogenate. After centrifugation, cells were resuspended in medium.

### 2.2 Cell Culture

Our culture methods refer to previously published studies [16, 17]. We used 1640 medium (Gibco) to culture MGS‐803, MKN‐28 and NCI‐N87 cell lines, and Ham's F‐12 (Gibco) to culture AGS. The cells were cultured with 10% fetal bovine serum (Gibco), 1% penicillin–streptomycin (Gibco). PDTCs were cultured in human GC organoids (GCOs) culture medium. For organoid culture, tumor cells were collected and seeded into Matrigel (082703, Mogengel, China) in the 96‐well cell culture plate (Costar) and added medium. We mixed Matrigel and the cells already mixed in base medium and added 10 μL drops to each well. Resuspension of cells in serum‐free medium and then mixing with Matrigel can better prevent adhesion and promote cell expansion. After the Matrigel solidified, a 60 μL cell line culture medium or GCOs culture medium was added to each well. The cell line medium contained 10% FBS and 1% penicillin–streptomycin. GCOs culture medium contained 10% R‐spondin1, 10% noggin conditioned medium, 50 ng/mL EGF, 10 mM Y‐27632, 50% Wnt conditioned medium, 1 nM gastrin, 200 ng/mL FGF 10, 2 mM TGFβi. For the 2D and spheroid culture, tumor cells were collected and placed on the 96‐well cell culture plate (Costar) and PrimeSurface 96 U plates (S‐bio, Japan). The formation of spheroid models relies on the technique of using the non‐adhesive surface. Every well contained about 1000 cells with 60 μL culture medium. Cell viability was evaluated every day by using the CellTiter‐Glo cell viability assay (Beyotime, Shanghai, China).

### 2.3 Drug Test

5‐FU (MCE, USA) and oxaliplatin (MCE, USA) concentration dilution was performed according to Table 1. Drugs were added on Day 3 and cell viability was evaluated using a CellTiter‐Glo assay (Beyotime, Shanghai, China) on Day 6. The concentration of each drug was adjusted to obtain in clinical pharmacokinetic studies (Table 1). The final concentrations for cell exposure are calculated in accordance with the molecular weight (MW), clinical dose indicated for GC (for 5‐FU and oxaliplatin), and exposure time (72 h). We used the half‐maximal inhibitory concentration (IC_50_) to measure the toxicity of a drug to a cell or the cell's ability to tolerate a drug.

**TABLE 1:** Drug concentrations applied.

Drug name	Clinical dose for gastric cancer (mg/m^2^)	Clinical dose for gastric cancer (mg/kg)	MW	Gradient dilution of drug concentration (μg/mL)				
				1	2	3	4	5
5‐Fluorouracil	500‐600	12–15	130.08	65.61	21.87	7.29	2.43	0.81
Oxaliplatin	130	—	397.29	196.83	65.61	21.87	7.29	2.43

### 2.4 Hypoxia Experiment

Add BBoxiProbe O91(Bestbio, Shanghai, China) to the 96‐well plate on Day 6. The fluorescent probe can enter the cell freely through the living cell membrane and react with the hypoxic injury products in the cell to form green fluorescent products. The green fluorescence increases with the degree of hypoxia and can be detected by fluorescence microscopy (Mshot, Guangzhou, China).

### 2.5 Reverse Transcription–Quantitative Polymerase Chain Reaction (RT–qPCR)

Target gene sequences were obtained from the NCBI database and primers were designed (Table 2). RNA was extracted with the MicroElute Total RNA Kit R6831 (OMEGA, China) and reverse‐transcribed into cDNA. RT–qPCR was performed on a QuantStudio 7 Flex real‐time PCR instrument with the SYBR Green kit (ThermoFisher, Fremont, CA, USA) using the corresponding primers (the sequences are shown in Table 2).18S was selected as the internal reference and the relative expression of target genes in different groups was expressed as 2^−ΔΔCT^.

**TABLE 2:** Primer sequences of genes.

Genes	Forward primer	Reverse primer
18S	CGACGACCCATTCGAACGTCT	CTCTCCGGAATCGAA CCCTGA
STAT1	AAGGACAAGGTTATGTGTATAG	CATTGGTCTCGTGTTCTC

### 2.6 Western Blotting

Proteins were extracted using RIPA lysis buffer supplemented with proteinase inhibitors (Roche, Basel, Switzerland). Samples were resolved in 12%–15% SDS‐PAGE and transferred onto PVDF membranes. Blocking was performed in 5% milk for 1 h and the membranes were incubated in primary antibodies overnight at 4°C. Membranes were incubated with HRP‐conjugated secondary antibody for 1 h and proteins were visualized using ECL substrate (ThermoScientific). The primary antibodies were PI3K (1:2000, 67 071‐1‐Ig, Proteintech), PTEN (1:2000 dilution, 22 034‐1‐AP, Proteintech), Cleaved PARp‐1(1:1000, #9102, Cell Signaling), GAPDH (1:4000, bsm‐0978M, Bioss).

### 2.7 H&E and IHC Staining Analysis

Cells were fixed in 10% formalin, embedded in paraffin, sectioned, and stained with hematoxylin and eosin (H&E). Anti‐Ki67 Rabbit monoclonal antibody (ServiceBio, Wuhan, China; cat. no. GB13030‐2, 1:200 dilution), Anti‐Caspase‐3 rabbit polyclonal antibody (ServiceBio, Wuhan, China; cat. no. GB11009‐1, 1:200 dilution), Anti‐Carbonic Anhydrase9/CA9 Rabbit polyclonal antibody (ServiceBio, Wuhan, China; cat. no. GB111184, 1:200 dilution), Anti‐MMP2 Rabbit polyclonal antibody (ServiceBio, Wuhan, China; cat. no. GB11130, 1:200 dilution), Anti‐MMP2 Rabbit polyclonal antibody (ServiceBio, Wuhan, China; cat. no. GB11132, 1:200 dilution), Anti‐E‐cadherin Mouse polyclonal antibody (ServiceBio, Wuhan, China; cat. no. GB12082, 1:200 dilution) and Anti‐Vimentin Rabbit polyclonal antibody (ServiceBio, Wuhan, China; cat. no. GB111308, 1:200 dilution) were used for the IHC analysis.

### 2.8 RNA‐Sequencing and Data Processing

As mentioned above, the two cell lines were cultured in three models, respectively. At least 3 biological replicates (including two lines and three models) were analyzed for each biological condition. Total RNA was extracted with the MicroElute Total RNA Kit R6831 (OMEGA, China) according to the manufacturer's instructions. RNA samples were submitted to quality control by RNA integrity number evaluation. Libraries for Illumina sequencing were constructed by poly A selection with the Illumina Kit. A paired end (2 × 100) run was performed.

### 2.9 Gene Expression Distribution and Principal Component Analysis

Due to the influence of sequencing depth and gene length, the gene expression value of RNA‐sequencing is generally expressed by FPKM instead of read count. FPKM has successively corrected the sequencing depth and gene length [18]. After calculating the expression value (FPKM) of all genes in each sample, we show the distribution of gene expression levels in different samples through a box diagram. The results abscissa in the figure is the name of the sample, and the ordinate is log2 (FPKM+1). The box diagram of each region has four statistics (the top and bottom are the maximum, upper quartile, median, lower quartile, and minimum). Principal component analysis (PCA) uses linear algebra to reduce dimension and extract principal components from tens of thousands of genetic variables. PCA analysis of gene expression values (FPKM) in all samples was performed.

### 2.10 Differential Expression Analysis and KEGG Enrichment Analysis

The original read count was first standardized, mainly to correct the sequencing depth. Then the statistical model calculates the hypothesis testing probability (P value) and finally conducts multiple hypothesis testing corrections to obtain the FDR value (error detection rate, *p* adj is its common form) [19, 20]. DEGs was screened between different sample groups using DESeq2 software, which satisfies |log2FC| ≥ 1 and *p* value ≤ 0.05 differential expression ranges to screen for differential genes between the two groups. Differential genes in all comparison groups were combined as differential gene sets. More than two groups of experiments can be used for cluster analysis of differential gene sets, and genes with similar expression patterns can be clustered together. We used the mainstream hierarchical clustering to perform cluster analysis on the FPKM value of genes and the homogenized row (*Z*‐score). ClusterProfiler software was used for KEGG pathway enrichment analysis of differential gene sets.

### 2.11 Statistical Analysis

Statistical analysis was performed using the GraphPad Prism 8.0. Student's *t*‐test (two‐tailed) was used to compare the difference between two groups of data. All data were represented as mean ± standard deviation (SD). *p* < 0.05 was considered statistically significant where *p < 0.05, **p < 0.01, ***p < 0.001, and ****p < 0.0001.

## 3 Results

### 3.1 Comparison of Growth and Morphological Characteristics in Different Models

**FIGURE 2:**
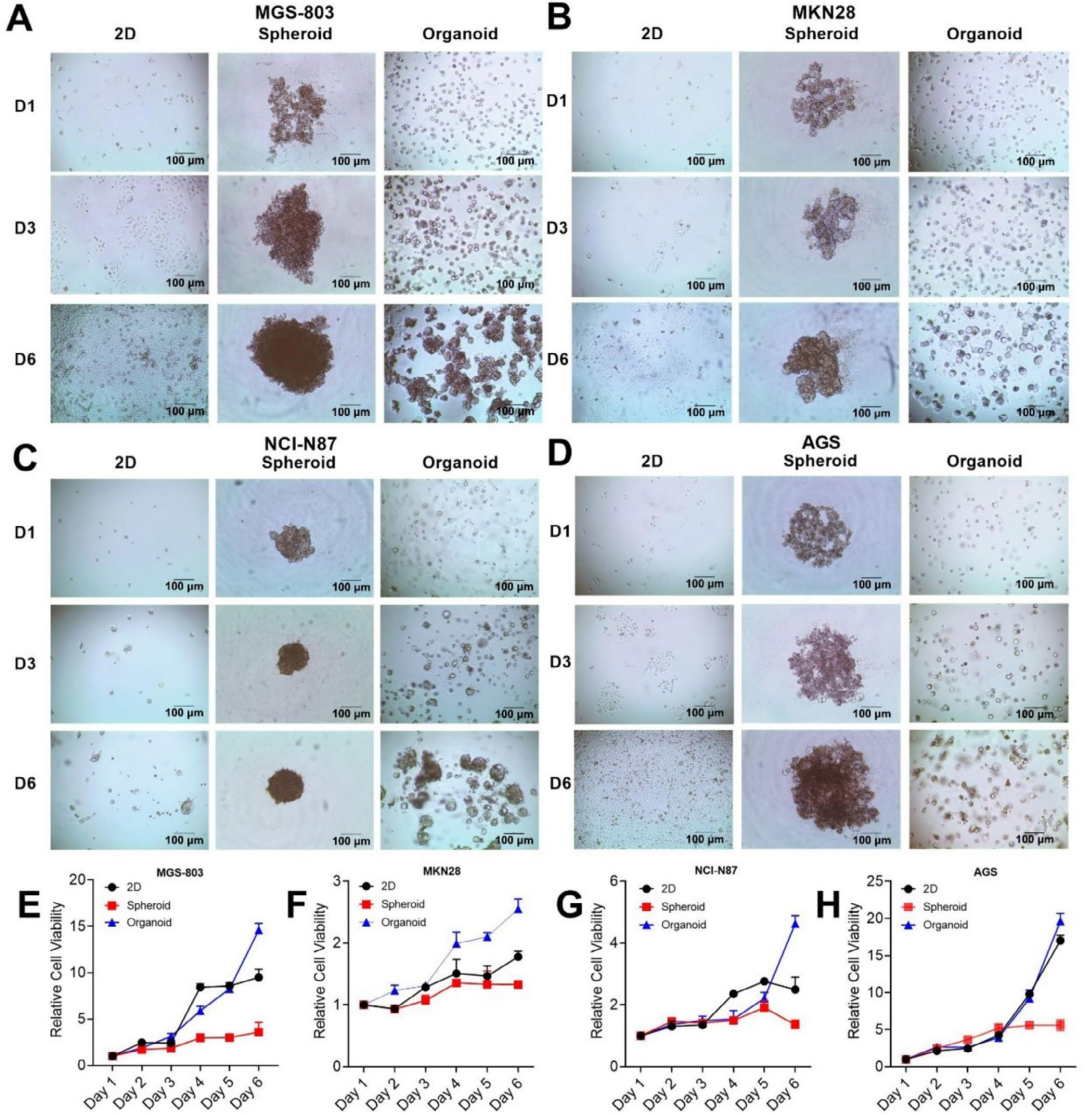
Comparison of growth and morphological characteristics in different models. One thousand cells were seeded to generate 2D cultures (adherent plastic plates), spheroids (non‐adherent plates), and organoids (embedded in Matrigel). MGS‐803 (A), MKN28 (B), NCI‐N87 (C), and AGS (D) growth were detected by microscopy on Days 1, 3, and 6 using a BZ‐X710 inverted microscope. MGS‐803 (E), MKN28 (F), NCI‐N87 (G), and AGS (H) cell viability were measured with the CellTiter‐Glo cell viability assay kit, normalized to the viability of Day 1. The cells were cultured with 10% fetal bovine serum, 1% penicillin–streptomycin, DMEM medium or 1640 medium. Data were representative of three independent experiments (Mean ± SEM).

**FIGURE 3:**
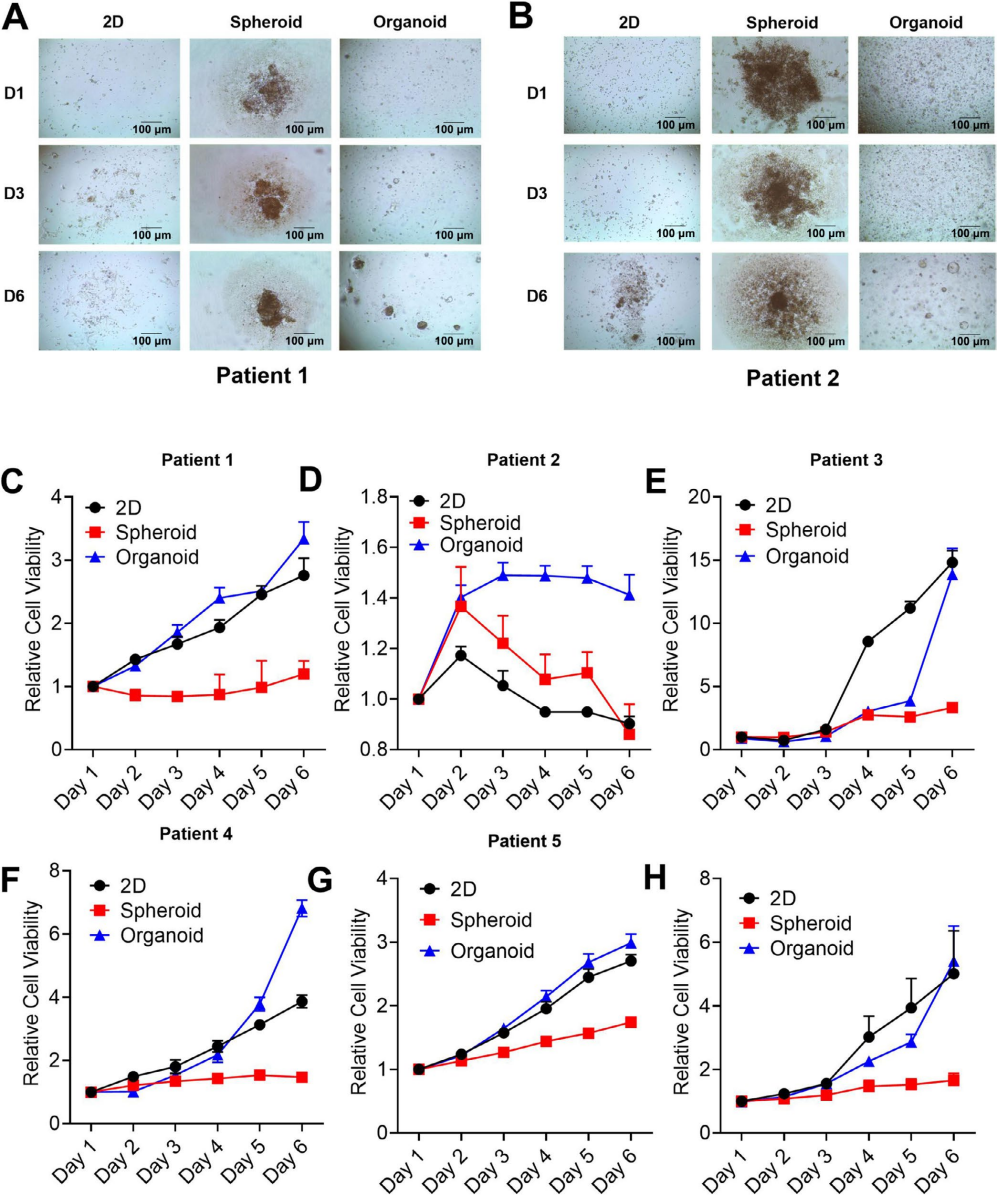
3D models facilitate PDTCs growth. GC tissue cells were treated by mechanical treatment and one thousand cells were seeded to generate 2D cultures (adherent plastic plates), spheroids (non‐adherent plates), and organoids (embedded in Matrigel). Patient 1–5 cell growth (A‐B) was detected by microscopy on Days 1, 3, and 6 using a BZ‐X710 inverted microscope. Patient 1–5 cell viability (C‐G) was measured with a CellTiter‐Glo cell viability assay kit, normalized to the viability of Day 1. The cells were cultured with human GC organoid medium. Data were representative of three independent experiments (Mean ± SEM). (H) represents the data of five patients combined.

We cultivated four GC cell lines to observe the shape and growth rate variations among the three models. Spheroids and organoids must have appropriate morphology and maintain continuous growth to achieve optimal state [21, 22]. First, we compared cell morphology and growth through microscopic observation and CTG measurements. All cell line culture results demonstrated success in forming spheroids and organoids, and were able to maintain growth in a medium containing standard serum (Figure 2A–D). The cell viability assay showed that the spheroid model had meager proliferation rates and a slower metabolic rate. Meanwhile, the organoids and 2D model had a fast growth rate (Figure 2F–H). Similarly, we seeded and cultured patient‐derived GC cells to further distinguish between primary cells and cell lines. We successfully cultured patient‐derived GC cells using the tissue digestion method. Spheroids and organoids were successfully formed, and the cells maintained their growth in the culture medium. (Figure 3A,B). Our results showed that patient‐derived GC cells were consistent with those of GC cell lines, with spheroids showing lower proliferation rates (Figure 3C–G). In contrast, the organoid model showed an increase in proliferation rate with time points (Figure 3C–G). In fact, organoids are wrapped in Matrigel. They are regulated by gradients and cell‐ECM mechanisms determined by cell–cell interactions and cell signaling [17]. Therefore, the growth characteristics of the model were not affected by cell lines (Figure 3H). In conclusion, we investigated the growth characteristics of the three models by culturing cell lines and PDTCs and demonstrated that both the organoid and 2D models exhibited high growth rates.

### 3.2 Differences in Drug Sensitivity of Different Models

**FIGURE 4:**
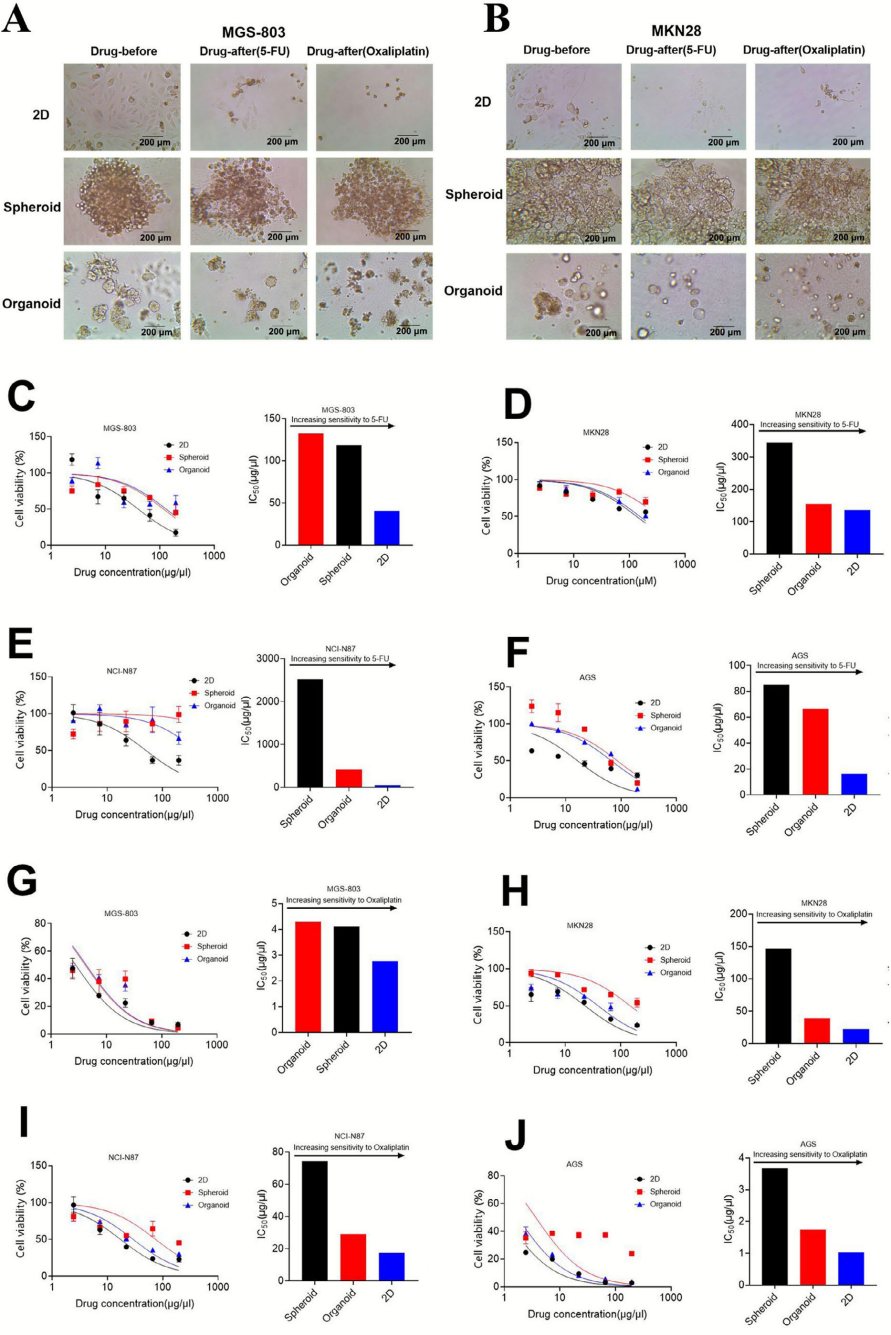
Differences in drug sensitivity of different models. MGS803, MKN28, NCI‐N87, and AGS were treated with increasing concentrations of 5‐FU and Oxaliplatin in three models for 72 h. The CellTiter‐Glo cell viability assay was performed to compare responses and viability was calculated as a percentage of the vehicle‐treated controls. Comparison of MGS803 (A) and MKN28 (B) untreated and maximum concentrations of 5‐FU and Oxaliplatin. Dose–response curve of MGS803 (C), MKN28 (D), NCI‐N87 (E), and AGS (F) in response to chemotherapeutic treatment with 5‐FU (*n* = 3, mean ± SEM). IC_50_ analysis of 5‐FU sensitivity in cell lines. Dose–response curve of MGS803 (G), MKN28 (H), NCI‐N87 (I), and AGS (J) in response to chemotherapeutic treatment with Oxaliplatin (*n* = 3, mean ± SEM). IC_50_ analysis of Oxaliplatin sensitivity in cell lines. The dose–response curve is a comparison of trends based on the range of values and data were representative of three independent experiments (Mean ± SEM).

**FIGURE 5:**
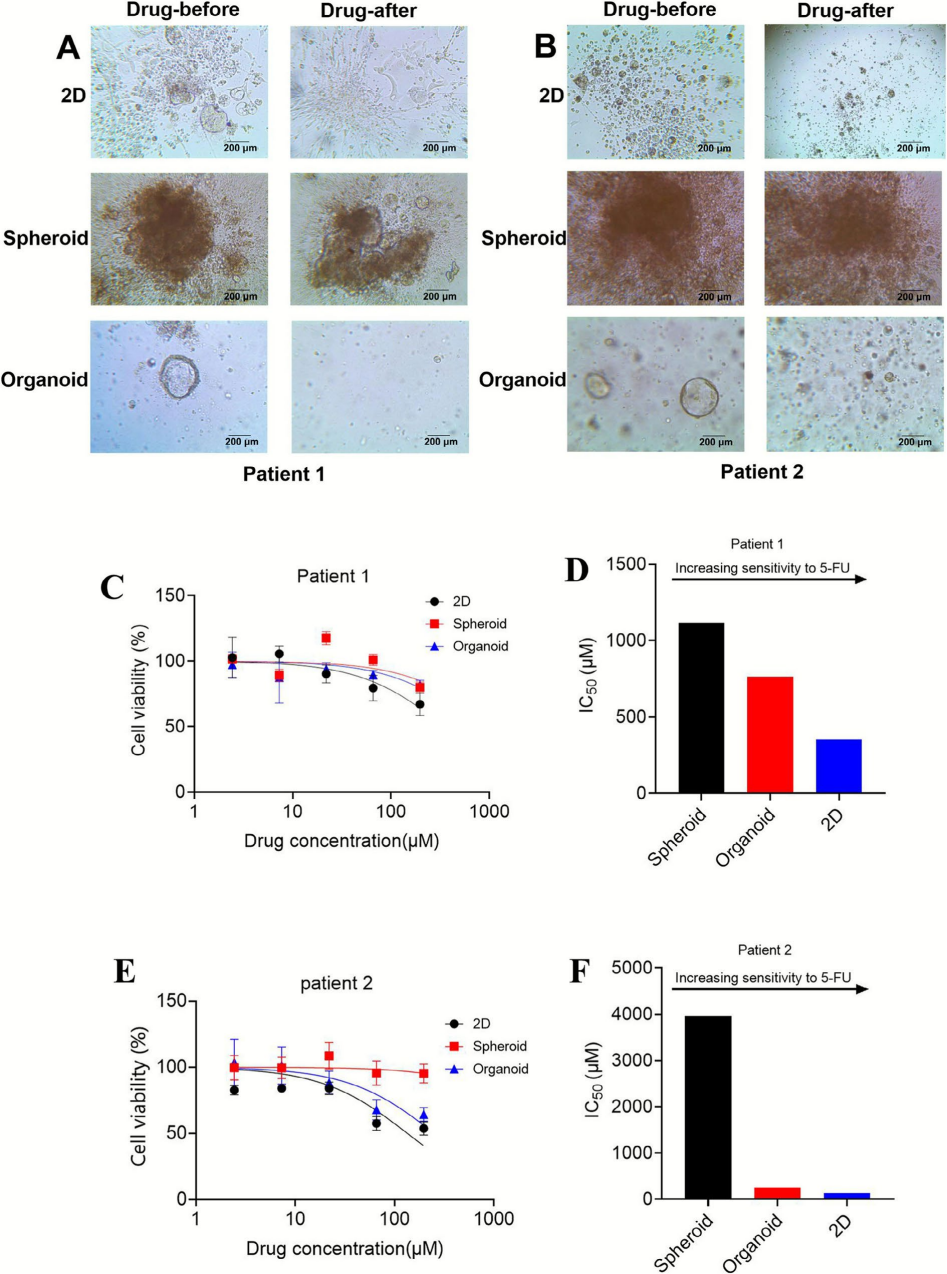
Drug sensitivity differences of PDTCs in different models. Patient1 and Patient2 were treated with increasing concentrations of 5‐FU in three models for 72 h. The CellTiter‐Glo cell viability assay was performed to compare responses and viability was calculated as a percentage of the vehicle‐treated controls. Comparison of Patient 1 (A) and Patient 2 (B) untreated and maximum concentrations of 5‐FU. Dose–response curve of Patient 1 (C) and Patient 2 (E) in response to chemotherapeutic treatment with 5‐FU (*n* = 3, mean ± SEM). IC_50_ analysis of 5‐FU sensitivity in Patient 1 (D) and Patient 2 (F). The dose–response curve is a comparison of trends based on the range of values and data were representative of three independent experiments (Mean ± SEM). After calculation, the relative cell viability of the three patients was significantly different.

5‐fluorouracil (5‐FU) and oxaliplatin are frequently used to treat GC, since oxaliplatin, either alone or in combination with 5‐FU, is more effective than cisplatin for treating advanced cancer [23, 24]. We investigated the responses of four GC lines to 5‐FU and oxaliplatin using three models. Through microscopic observations, we detected differences in the drug responses to 5‐FU and oxaliplatin among all GC cell lines (Figure 4A,B). The CTG test results also revealed that GC cells were sensitive to 5‐FU and oxaliplatin, and we calculated the IC_50_ of the three models based on the drug sensitivity curve (Figure 4C–J). As mentioned above, we designed the drug according to Table 1. The spheroid and organoid model demonstrated higher cellular resistance to drugs, whereas the 2D model exhibited lower resistance among the three models (Figure 4C–J). The same trend was observed in the comparative experiments of models through the cultivation of PDTCs (Figure 5C–H). Specifically speaking, we tested the drug response of PDTCs to 5‐FU. The results showed that these cells from patients were smaller and less active after dosing than before dosing by microscopic observations (Figure 5A,B). Additionally, the CTG test results revealed that PDTCs were sensitive to 5‐FU (Figure 5C–F). We have summarized the collected patient data and experimental results in Table 3. These findings imply that patient tissue‐derived cells serve as an essential and efficient tool for predicting drug sensitivity response. When the drugs were effective, the spheroid and organoid models exhibited greater resistance to these drugs compared with the 2D model.

**TABLE 3:** Patients' baseline characteristics and drug susceptibility testing to 5‐FU in vitro.

	Sex	Age	TNM stage	Drug susceptibility testing in vitro (5‐FU)
Patient 1	Male	53	II–III	Sensitive
Patient 2	Male	72	II–III	Sensitive
Patient 3	Male	46	III	Resistant
Patient 4	Male	52	III	Sensitive
Patient 5	Male	58	III	Sensitive

### 3.3 Comparison of Pathophysiological Characteristics in Different Models

**FIGURE 6:**
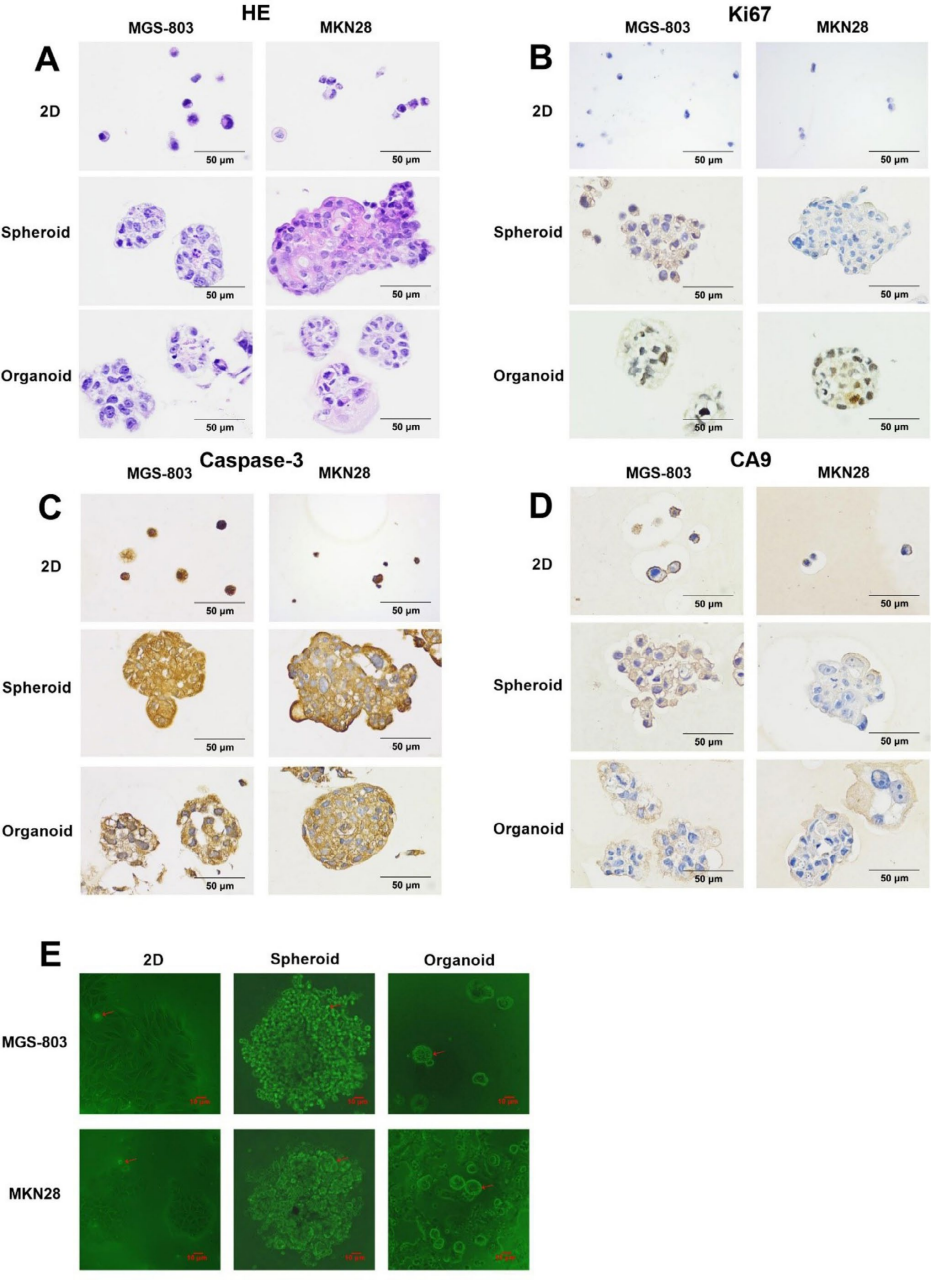
Comparison of pathohistological characteristics in different models. MGS803 and MKN28 (seeded with 1000 cells) were cultured for 6 days in three models (2D, spheroid and organoid). Histological findings in the three models of MGS‐803 and MKN28 (A) (H&E staining; scale bar = 50 μm). MGS‐803 and MKN28 were stained with anti‐rabbit Ki67 antibody (B), anti‐rabbit Caspase‐3 antibody (C) and anti‐rabbit CA9 antibody (D) using IHCs (scale bar = 50 μm). MGS803 and MKN28 (E) were combined with BBoxiProbe O91 probe to observe green fluorescence intensity using a fluorescence microscope. The BBoxiProbe O91 probe was diluted with serum‐free medium at 1:1000. Data were representative of two independent experiments.

To investigate differences between the histopathological characteristics of the three models, we cultured the MGS‐803 and MKN28 cell lines. The cells in the 2D model were readily dispersed into single cells (Figure 6A). Meanwhile, the organoid and spheroid models formed histological structures: cells firmly contacted with a more intricate structure (Figure 6A). We next investigated whether the distribution and expression of common markers, such as the proliferation protein Ki67, the apoptotic protein Caspase3, and the hypoxia protein CA9, were affected using different models. Most 3D models displayed high Ki67‐positive cells (Figure 6B). Caspase3 was expressed in all models, with the highest expression in the 2D model (Figure 6C). According to IHC staining results, higher CA9 protein expression was observed in organoids and spheroids, and lower CA9 protein was expressed in the 2D model (Figure 6D). The BBoxiProbeO91 fluorescent probe reacts with intracellular hypoxia injury to produce green fluorescence. We analyzed the spatial distribution of cells in 2D and 3D models by generating green fluorescence in living cells. The results showed that the hypoxic region of the spheroid model was the most significant, followed by the organoid model and then the 2D model (Figure 6E). These results may be one of the reasons why 3D models are more susceptible to drug resistance.

### 3.4 The Organoid Model Enhances the Activation of the PI3K/PTEN Pathway

**FIGURE 7:**
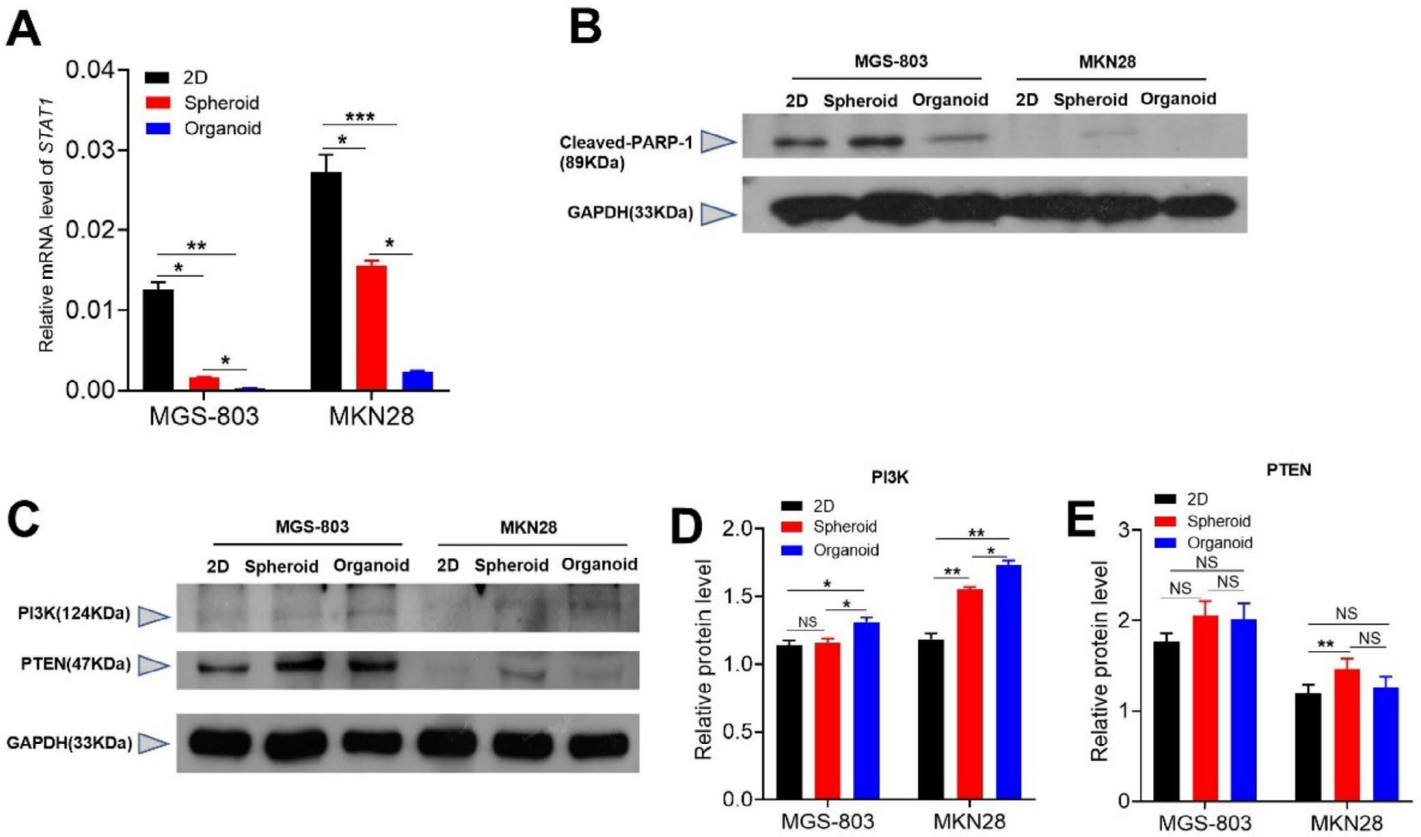
The organoid model enhances activation of the PI3K/PTEN pathway. Genes were extracted from MGS‐803 and MKN28 that were cultured for 6 days in three models, and then the gene expression of STAT1 (A) was determined by RT‐qPCR. Proteins were extracted from MGS‐803 and MKN28 that were cultured for 6 days in three models, and then the levels of Cleaved‐PARP‐1 (B), PI3K (C), and PTEN (C) were determined by Western blot. The density of the PI3K (D) and PTEN (E) Western blot band intensity was quantified by Image J. Data were representative of three independent experiments (Mean ± SEM). Data were analyzed by Two‐way ANOVA at each model. **p < 0.01; ****p < 0.0001; NS, not statistically significant.

Cancer is a progressive disease that undergoes multiple stages, each associated with specific molecular, genetic, and cellular alterations that allow it to acquire an increasingly malignant phenotype. This evolutionary process would not occur without continued cell proliferation and cell survival [25]. By detecting the expression of genes and proteins, we explored the changes in the activation levels of the migration and differentiation signaling pathways in three models. Studies have shown that activation of *STAT1* can promote cell apoptosis and inhibit cell proliferation [26]. The quantitative RT‐PCR results showed that the expression of *STAT1* mRNA in the organoids was lower than that in the other two models (Figure 7A). The transcriptional activation of caspase‐3 by STAT1 could induce apoptosis [27, 28]. The IHC staining results showed that the organoid model had the lowest expression of caspase‐3 (Figure 7C). In addition, cleaved PARp‐1 is an essential indicator of apoptosis and is generally considered an indicator of caspase‐3 activation [29]. The Western blot results showed that cleaved PARp‐1 expression was lowest in organoids compared with the 2D model and spheroids (Figure 7B). Abnormal regulation of the PI3K/AKT pathway further promotes tumor cell proliferation and is closely associated with tumor invasion and metastasis [30]. However, PTEN can negatively regulate PI3K activation [31]. The Western blot results showed that the organoid model had high PI3K protein expression (Figure 7C,D). In addition, PTEN expression was lower in the 2D and organoid models (Figure 7C,E). These findings imply that the PI3K/PTEN signaling pathway can be markedly activated in the organoid model, while the production of apoptosis‐related proteins is greatly inhibited.

### 3.5 3D Models Promote Cancer Cells With EMT Progress

**FIGURE 8:**
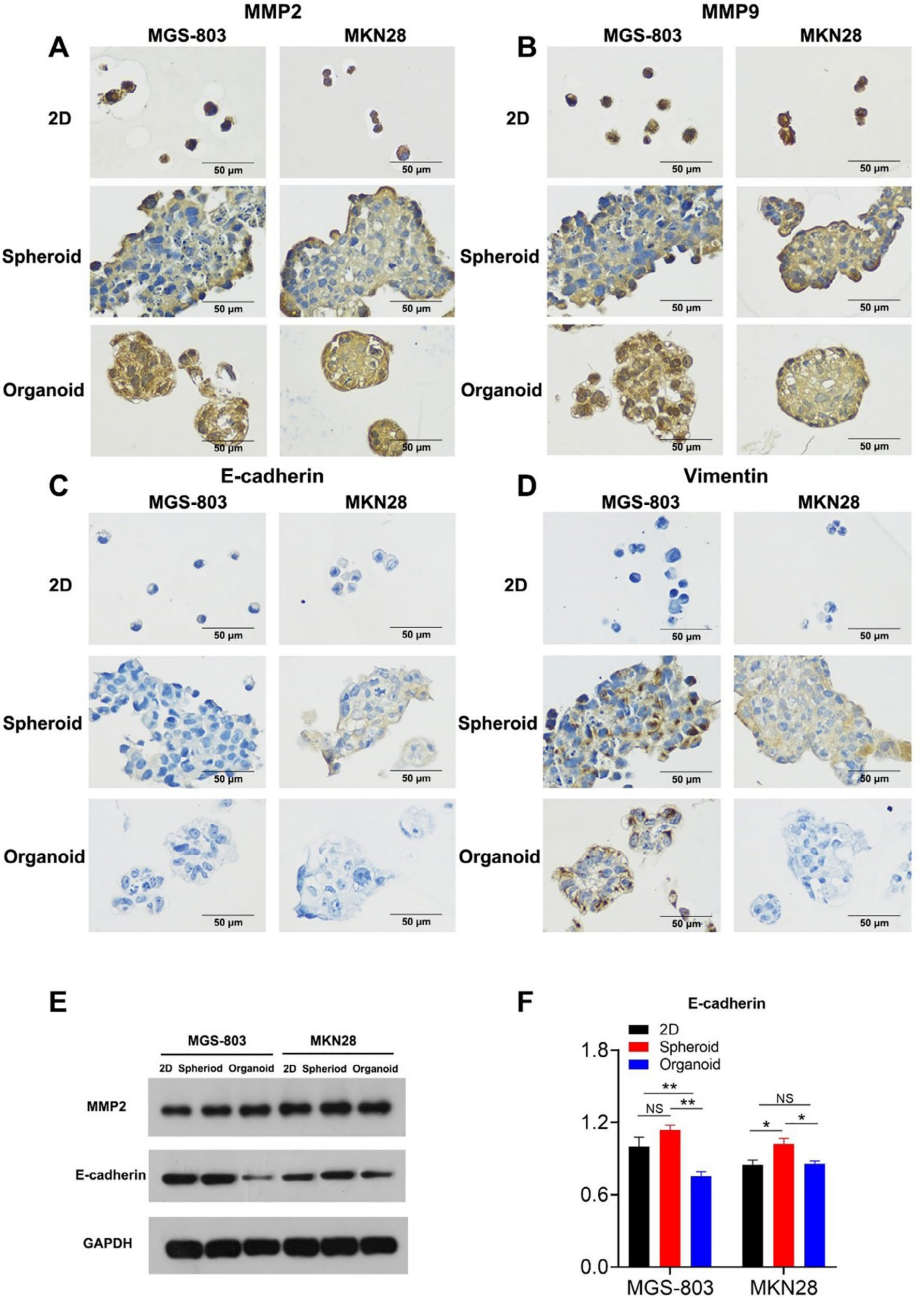
The Culture of 3D Models Promotes EMT Progress. MGS‐803 and MKN28 were stained with anti‐rabbit MMP2 antibody (A), anti‐rabbit MMP9 antibody (B), anti‐mouse E‐cadherin antibody (C) and anti‐rabbit Vimentin antibody (D) using immunohistochemistry methods (scale bar = 50 μm). Protein was extracted from MGS‐803 and MKN28 that were cultured for 6 days in three models, and then the levels of MMP2 (E) and E‐cadherin (E) were determined by Western blotting. The density of MMP2 (E) and E‐cadherin (E) band intensity was quantified by Image J. Data were representative of two independent experiments. Data were representative of three independent experiments (Mean ± SEM).

To further explore the differences in metastasis and invasion among the three models, we examined the expression levels of MMP2 and MMP9, molecules involved in tumor metastasis. MMP2 and MMP9 were substantially expressed in all models, especially the organoid model (Figure 8A,B). The Western blotting results also show that MMP was higher expressed in the organoid model (Figure 8E). Due to the normal epithelial multilayered cellular structure impeding the metastasis and invasion of malignant tumors, tumor cells undergo EMT alterations to lose their epithelial phenotype and gain motility [32, 33, 34]. To determine whether the differences in the model's metastatic and invasive activities were related to the transformation of EMT, we detected the expression of the epithelial cell surface marker E‐cadherin and the mesenchymal cell surface marker vimentin. Since the loss of E‐cadherin can induce tumor cell differentiation and high metastasis, we found that E‐cadherin expression was low in all models by IHC analysis, especially the organoid model (Figure 8C,D). Vimentin, in contrast to E‐cadherin, was expressed in the 3D models (Figure 8C,D). The Western blotting results also showed that E‐cadherin was lowest expressed in the organoid model (Figure 8E,F). To demonstrate the reliability of the experiment, we supplemented the positive and negative controls with immunohistochemistry (Figure S1). Our research suggested that the 3D models induced cell differentiation more effectively than the 2D model, possibly because the former promotes cell–cell interactions.

### 3.6 Gene Expression Analysis of Different Cells in Different Models

**FIGURE 9:**
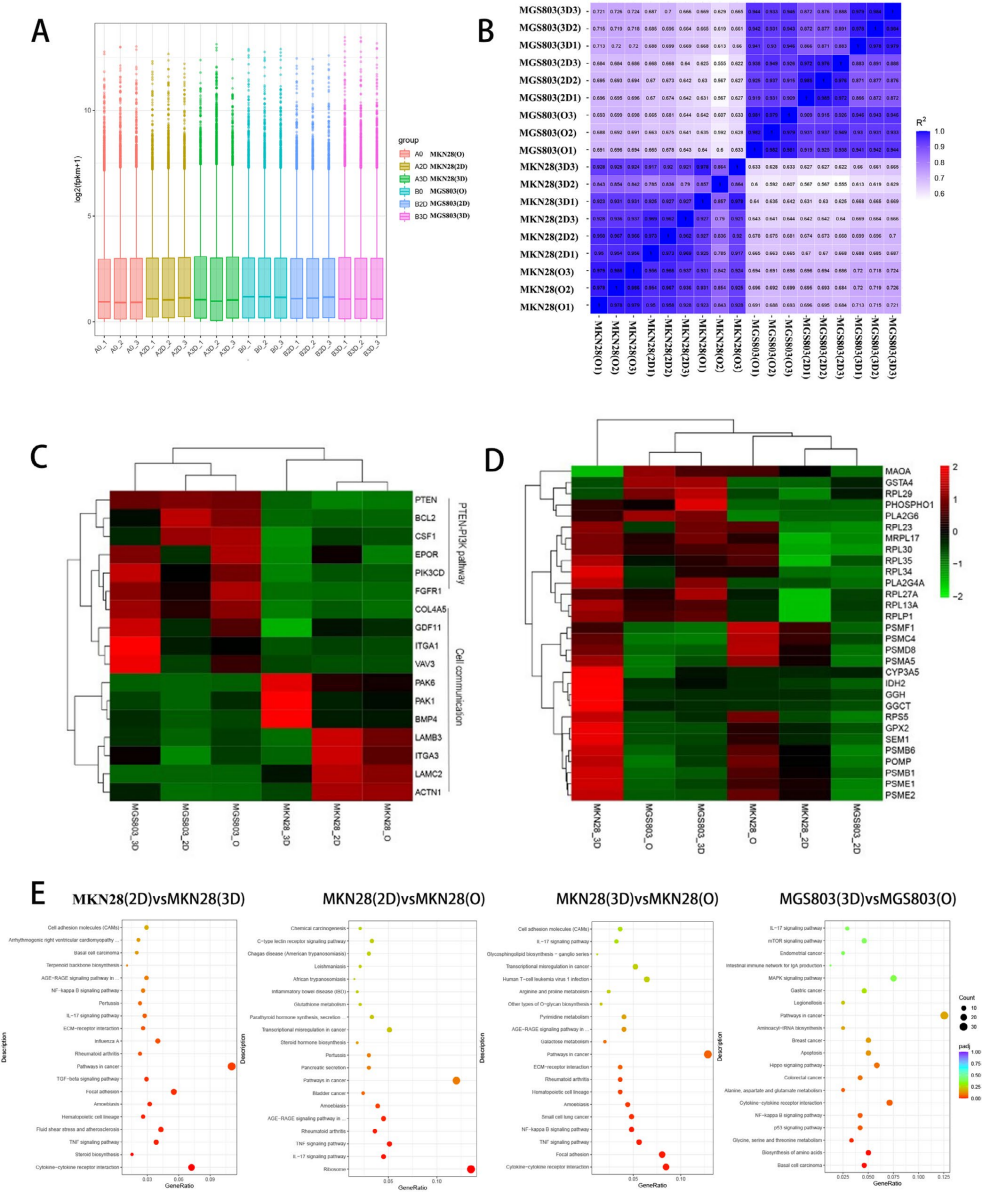
Gene expression analysis of different cells in different models. RNA was extracted from MGS‐803 and MKN28 that cultured 6 days in three models, and then RNA‐sequencing analysis was performed. PCA plot of RNA‐sequencing data of two cell lines (MKN‐28 and MGS‐803) in three models (A). Pearson correlation between samples data of two cell lines in three models (B). Heatmaps show the expression of representative signaling pathway gene clusters that reflect key molecular characteristics of two cell lines of three models. The Log2TPM value of each gene was used for clustering after mean centering. Upregulation is shown in red, while downregulation is shown in green (C). KEGG enrichment was used to analyze the related functions of the two cell lines. Downregulation is shown in red, while Upregulation is shown in green (D). Data were representative of three independent experiments (Mean ± SEM) (E). KEGG pathway enrichment analysis showed significant differences between MKN28 (2D) model and MKN28 (3D and O) models, as well as between MKN28 (3D) model and MKN28 (O) model.

We conducted RNA sequencing on cells derived from a total of two cell lines (MKN28‐2D and MGS‐803) to further explore the differences among the models at the molecular level. The gene expression distribution result showed that the findings were consistent and repeatable (Figure 9A). Pearson correlation between samples had a high repeatability (Figure 9B). According to the RNA sequencing results, numerous genes and signaling pathways, such as the PTEN‐PI3K signaling pathway, cellular communication, ribosome biosynthesis, and proteasome biosynthesis, showed significant differences among the three models (Figure 9C,D). The heat map result showed that molecules related to the PTEN‐PI3K signaling pathway, such as PTEN, BCL2, CSF1, EPOR, PIK3CD, and FGFR1 were substantially expressed in the MGS‐803 organoids (Figure 9C). To further explore whether drug resistance in 3D culture is related to biosynthesis and metabolism, we analyzed proteasome (SEM1, PSMB1, PSMB6, PSME1, PSME2, PSMF1, PSMA5, PSMD8, PSMC4, POMP), ribosome (RPLP1, RPL13A, RPL23, RPL29, RPL35, RPL27A, RPL30, RPL34, RPS5, MRPL17) and other metabolic pathway‐related molecules (GSTA4, GGCT, IDH2, GPX2, PA2G4A, PLA2G6, PHOSPHO1, CYP3A5, MAOA, GGH) (Figure 9D). We found that the spheroid model had a greater number of molecules related to biosynthesis and metabolism with high expression levels, followed by the organoid model and then the 2D model. This differential expression may reveal one of the reasons why 3D models, such as the spheroid and organoid models, are highly resistant to drugs. (Figure 9D). The KEGG pathway enrichment analysis showed that the pathways in cancer and cytokine‐cytokine receptor interaction pathway were significantly different between the spheroid model and the organoid model (Figure 9E). Pathways in cancer and cytokine‐cytokine receptor interaction pathway were significantly different between the MKN28(2D) model and the MKN28(3D) model (Figure 9E). Pathways in cancer and ribosome pathway were significantly different between the MKN28(2D) model and the MKN28(O) model (Figure 9E). These results imply some potential critical pathways for us in these models.

## 4 Discussion

This study aimed to reveal disparities in the growth characteristics and drug sensitivity evaluation of GC cells in 2D and 3D cultures and probe into potential mechanisms. In our study, we demonstrated that GC cell lines and PDTXs formed spheroids and organoids and indicated that the growth of spheroids and organoids both accelerated over time. Therefore, the cells in the three models proliferated at different rates. Moreover, the drug responses varied among the three models. The drug sensitivity experiment results showed that spheroids and organoids, especially spheroids, had high drug resistance in the three models. Our results clearly indicate that the sizes of organoids and spheroids differ significantly. The size factor plays a crucial role in characteristics such as drug resistance and growth rate. These 3D models exhibited protein expression and spatial distributions of marker molecules that were close to those in vivo tumors. The markers included those related to proliferation, apoptosis, and hypoxia. These findings suggest that the processes of proliferation, apoptosis, and hypoxia in 3D models have an impact on drug resistance and growth rate.

We endeavored to elucidate the mechanisms underlying their drug resistance at the molecular level. It was discovered that the organoid model exhibited the lowest expression level of cleaved PARP and a relatively higher expression level of the PTEN‐PI3K pathway. The activation of the PTEN‐PI3K signaling pathway implies that the cells within the organoid model are in a state of active growth. Our RNA‐sequencing analysis results suggest that this may be due to higher expression of biosynthesis and metabolism‐related molecules in spheroids and organoids. In addition, spheroids had the highest resistance to drugs in most cases, as demonstrated by primary cells from patient tissues. In addition to the mentioned reasons, we guess there are other reasons: (i) the way the drug enters the cell; (ii) the intricate microenvironment of the 3D model; (iii) the larger surface of the 2D model for drug contact; (iv) difficulty for the drugs to access the cell nuclei in the 3D model; (v) uneven gene and protein activation/expression across the three models; (vi) different growth rates amongst the three models; and (vii) complex PDTC medium composition.

In some scenarios, 3D models have the potential to render preclinical research data more robust and reliable, reduce the necessity for animal testing, and facilitate the translation of these research findings into clinical practice. However, despite their closer resemblance to clinical tumor tissue, a comprehensive understanding of the heterogeneity and pathogenesis of human cancers remains considerably distant. Furthermore, these models lack the intricacy of the immune system, angiogenesis, and fibroblasts found in vivo, which are necessary to assess the organ‐damaging effects of drugs [35].

Cancer cell lines cultured in 2D on plastic surfaces are regarded as incapable of precisely mimicking the in vivo tumor microenvironment [36, 37, 38, 39, 40, 41]. Our research suggests that more representative 3D models than 2D models not only enhance tumor metabolic progress but also strengthen cell‐to‐cell connections. During tumor development, the structure and composition of the ECM undergo dramatic changes, often enabling cellular transformation, inflammation, invasion, metastasis, and angiogenesis [42, 43]. These changes in the tumor ECM result in the activation of cellular pathways such as Rho/Rock PTEN and PI3K‐AKT [44, 45]. In summary, we conducted demonstrations of the existence of similarities and differences among the three models by culturing cell lines and cancer tissue‐derived cells. Based on experiments with gene and protein assays, gene sequencing, and drug sensitivity testing, we found that the differences between the models may involve activation of the PTEN/PI3K‐AKT pathway, cancer‐related pathways, and synthetic and metabolism‐related molecules. We are also focused not just on drug sensitivity and related apoptotic signaling pathways. We believe that the differences between the models cause differences in cell morphology mainly by the culture medium and the culture method and depending on their characteristics lead to how the cells in that model are better selected for activation of relevant signaling pathways, thus affecting the expression and secretion of relevant proteins. As a result, the model can be further refined by identifying pertinent signaling pathways, leading to better modeling of research areas of interest.

Nevertheless, our study is not without its limitations. Despite our strict compliance with the method for culturing 3D cells as proposed in the published article and the successful execution of the study, the process of constructing and culturing 3D models remains intricate and lacks standardization [37, 38, 39, 40, 41]. Since there is no unified industry standard for GC organoid modeling currently, we ensured the study's reproducibility via the following measures: strict control of culture conditions (e.g., 37°C temperature, 5% CO_2_ concentration, medium replacement every 48 h); and three technical replicate validations for key steps (e.g., tissue digestion time, Matrigel concentration) with a coefficient of variation < 10%. In subsequent studies, we will further explore a standardized process based on this protocol. Secondly, the cellular composition and microenvironmental constituents within the 3D models diverge substantially from those of authentic human tissues, thereby hampering their comprehensive exploitation in emulating complex biological processes [38]. We cultured the 3D model by referencing relevant literature and optimized it to “simulate the in vivo microenvironment”; for example, VEGF and TNF‐α were added to the GC organoid medium to mimic the tumor microenvironment. However, we acknowledge this model lacks immune cells (e.g., T cells, macrophages) and vascular structures, so it cannot fully recapitulate in vivo tumor‐microenvironment interactions—an inherent limitation of this study. In future research, we can establish a “GC organoid‐peripheral blood mononuclear cell (PBMC)” co‐culture system. By introducing the immune microenvironment, we will further verify this study's drug sensitivity results. Meanwhile, combining single‐cell sequencing technology will enable analysis of the interaction mechanisms between the microenvironment and tumor cells to refine the drug resistance theory. The sample size of this study was comparatively small, a factor that has the potential to impinge upon the statistical validity and generalizability of the results. The sample size of PDTC is relatively small, limited by three objective factors: challenging clinical acquisition of fresh surgical specimens from GC patients (which requires informed consent from patients), restrictions on using “clinical specimens for in vitro modeling” as specified in ethical approval, and high difficulty in establishing patient‐derived organoid models (which demand a large number of fresh surgical specimens). Despite the small sample size, the selected samples have complete information, thus ensuring the samples' representativeness to a certain extent. Even though we discerned some significant disparities, an enlarged sample size would be instrumental in validating the reliability of these findings. Thus, the sample size of PDTC in this study is relatively small, which may reduce the statistical power of some conclusions and make it difficult to rule out the interference of individual differences. Therefore, the external generalizability of the conclusions requires verification in a larger sample cohort. For subsequent studies, collaboration with gastrointestinal surgery departments across multiple centers can be pursued to expand the PDTC sample size to more than 50 cases. Meanwhile, by integrating patients' postoperative follow‐up data, a verification system for “model‐clinic” correlation can be established to enhance the clinical translation value of the conclusions. Consequently, the functional validation and data analysis of 3D models prove to be more arduous than those of 2D models and necessitate the development of more sophisticated techniques and methodologies. Moreover, the paucity of functional validation experiments circumscribes our capacity to interpret the observed biological significance. Future investigations ought to be oriented towards optimizing the culturing conditions of 3D models, integrating diverse cell types and biomaterials, and devising high‐throughput functional validation techniques to enhance the physiological pertinence and application value of these models. In conclusion, the refinement of the 3D culture system as a platform for drug screening is worth advancing. Secondly, in future research endeavors, we ought to concentrate on elucidating its underlying molecular mechanisms to clarify the biological significance of these differences and explore its potential value as a pre‐clinical platform model.

## Author Contributions

**Wenhui Zheng:** formal analysis (equal), writing – original draft (equal). **Yubiao Lin:** formal analysis (equal), writing – original draft (equal). **Lulin Ji:** formal analysis (equal), writing – original draft (equal). **Lihua Feng:** formal analysis (equal), writing – original draft (equal). **Xin Fan:** formal analysis (equal), writing – original draft (equal). **Zhigao Zheng:** validation (equal). **Yingqin Gao:** validation (equal). **Kaida Huang:** validation (equal). **Guoqin Qiu:** funding acquisition (equal), investigation (supporting), resources (equal). **Yide Chen:** funding acquisition (equal), investigation (supporting), resources (equal). **Fanghong Luo:** conceptualization (equal), funding acquisition (equal), investigation (lead), resources (equal). **Shuitu Feng:** conceptualization (equal), funding acquisition (equal), investigation (lead), resources (equal).

## Funding

This work was supported by funds from the Natural Science Foundation of Fujian Province (No. 2023D007); the Science and Technology Program of Haicang District of Xiamen, China (Nos. 350205Z20232012, 350205Z20222005, 350205Z20222004).

## Conflicts of Interest

The authors declare no conflicts of interest.

## Supporting information


**Figure S1:** Positive and negative controls of E‐cadherin and vimentin.

## Data Availability

Data will be made available upon reasonable request.
