# Supplementary figures and images for: Comparison of Gastric Cancer Models Using Different Dimensions In Vitro
Source: Cancer Rep (Hoboken). 2025 Nov 27;8(12):e70401. doi: 10.1002/cnr2.70401 (PMC12659928; doi:10.1002/cnr2.70401)

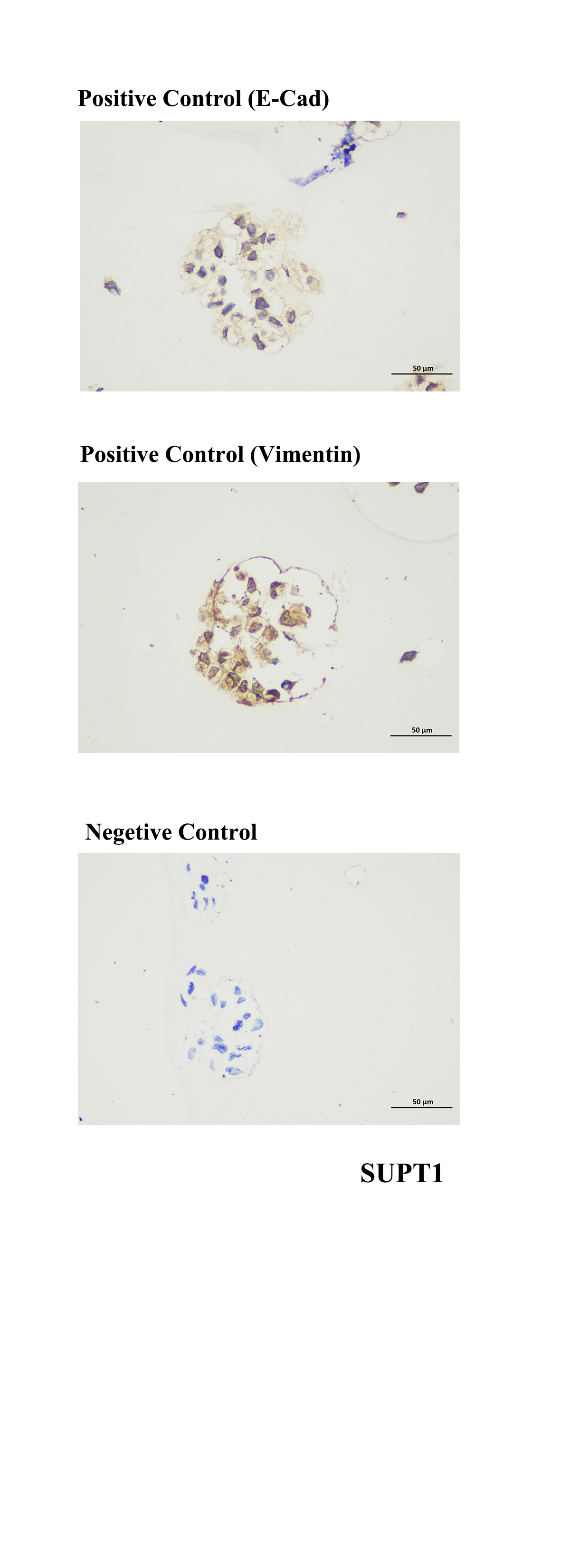

Supplement: Supplementary file 1 — Figure S1: Positive and negative controls of E‐cadherin and vimentin. [file CNR2-8-e70401-s001.jpg]
